# Spectrum of disease in HIV-positive patients presenting to a tertiary care hospital: a retrospective, cross-sectional review in Kumasi, Ghana

**DOI:** 10.1186/s12879-018-3332-1

**Published:** 2018-08-23

**Authors:** Richard Odame Phillips, Alexis Steinmetz, Justin Nichols, Emmanuel Adomako, Emmanuel Ofori, Emilia Antonio, St.-Martin Allihien, Collins Peprah-Addae, William Adams

**Affiliations:** 1 Komfo Anokye Teaching Hospital, Kumasi, Ghana; 2 Kwame Nkrumah University of Science and Technology, Kumasi, Ghana; 3 University of Rochester Medical Center, Rochester, NY USA; 4 Lewis Katz School of Medicine at Temple University, Philadelphia, PA USA; 5 Clinical Research Office (CRO), Loyola University Chicago Health Sciences Division, Chicago, USA

**Keywords:** HIV, AIDS, Global Health, Ghana, Epidemiology

## Abstract

**Background:**

HIV remains a significant public health dilemma in West and Central Africa. HIV-related morbidity and mortality are unjustly high, yet little is known about the spectrum of complicating comorbidities in HIV-positive patients who are admitted to hospitals in these regions.

**Methods:**

This study involved a retrospective chart review to determine the common comorbidities and mortality rate of HIV-infected patients admitted over a six month period to the internal medicine service at the Komfo Anokye Teaching Hospital (KATH), a tertiary care center in Ghana. Patients admitted with a known or new HIV diagnosis from January to July 2016 were included. Data were collected regarding the number of new versus known cases admitted, the most common presenting complaints, final admitting diagnoses, and causes of mortality in these patients.

**Results:**

During the six-month study period, 250 HIV-positive patients were admitted to KATH, and 245 of these individuals had valid survival time recorded. Of these patients, 145/245 (59.2%) were female. Median age of patients included in the study was 42 years old (IQR 35–51). The mortality rate for HIV patients admitted to the hospital was 35.5% (87 patients). One hundred and forty-five (59.4%) patients had a known history of HIV documented in their patient charts, while the remaining patients were newly diagnosed with HIV during their inpatient stay. Pulmonary tuberculosis predominated among diagnostic findings, with 40.4% of HIV-infected patients diagnosed with the condition while admitted. Patients presenting with neurological symptoms on admission were 2.14 (95% CI: 1.27–3.61) times more likely to die than those without neurological symptoms (*p* = .004).

**Conclusions:**

Over 40% of HIV-positive patients admitted to KATH were newly diagnosed with HIV at admission. While pulmonary tuberculosis was the most common co-morbidity, patients presenting with neurological symptoms were at higher risk of death. This study suggests that enhanced outpatient screening is needed for early diagnosis and prompt HAART initiation, as well as increased access to diagnostic modalities and treatment for HIV-positive patients with neurological symptoms.

## Background

Although new HIV infections have declined significantly in western and central Africa in the last decade, these regions still account for 22% of total global infections [1]. The prognosis for persons living with HIV has improved dramatically since the advent of highly active antiretroviral therapy (HAART) [2], but these improvements have lagged behind in resource-poor countries [3]. Of the staggering 6.1 million people living with HIV in West and Central Africa, only 35% were accessing antiretroviral therapy in 2016 [1]. As a result, HIV-related morbidity and mortality remain unjustly high in this region [3–7]. Patients continue to present to inpatient care with WHO Class III and IV illness and undiagnosed or untreated HIV infection as a result of insufficient case finding, poor linkage to care and treatment failure or default [3, 4, 8–12]. This undoubtedly contributed to the 310,000 AIDS-related deaths that were reported in 2016 in western and central Africa [1]. In the nation of Ghana in the geographic region of West Africa, 230,000 adults were living with HIV in 2015 [13], but in this population there are currently limited morbidity and mortality studies of HIV-positive adults presenting to hospital.

Several studies in other nations have attempted to evaluate causes of hospitalization in HIV-infected patients and their associated mortality in resource-poor countries in order to better characterize these disparities [7, 11, 14–17]. The results are not surprising: most patients present with late stage HIV (WHO Classes III and IV), many have associated tuberculosis morbidity and resultant in-hospital mortality is high [3, 7, 14–16]. Regional differences in HIV co-morbidities have been noted within the African continent. For example, in East Africa, *Cryptococcus neoformans* infection continues to be a major burden of disease in immunocompromised patients [10]; however, the prevalence of cryptococcal antigenemia may be quite low in West Africa [18]. Thus, these studies are needed globally to identify the common HIV-related comorbidities on a regional basis. Epidemiological knowledge about disease presentations can inform better allocation of resources towards the diagnosis and treatment of common clinical presentations.

There are still advances in care that must be pursued in light of global targets in the HIV pandemic. The 90–90-90 goals advocate for substantial increases in case detection, treatment and follow-up care amongst this patient population, with goals of reaching 90% case diagnosis, 90% of patients diagnosed receiving antiretroviral treatment, and 90% of those on treatment achieving undetectable viral loads by the year 2020 [19]. In addition, the INSIGHT [20] and TEMPRANO [21] trials have led to the WHO’s 2015 recommendations for initiation of ART in all patients regardless of CD4 count [22].

In light of these recommendations and targets, our aim was to investigate the rate of known versus new HIV diagnosis as well as morbidity and mortality outcomes for HIV-positive patients admitted to the internal medicine department at the Komfo Anokye Teaching Hospital (KATH) in order to contribute to the development of strategies to improve care of individuals with HIV infection in Ghana.

## Methods

### Study design and population

This study took place at the Komfo-Anokye Teaching Hospital (KATH), a 1200-bed tertiary hospital located in Kumasi, the capital city of the Ashanti Region of Ghana. Patients are referred to KATH from each of the ten regions of Ghana. Adult patients greater than 18 years old admitted to the internal medicine service at KATH with documented HIV infection were included in the study. The study included patients who were admitted to the hospital with a previous diagnosis of HIV, as well as those who were newly diagnosed during their current admission. Patients without valid dates of admission, discharge or death recorded in the patient chart were excluded from analysis.

### Procedures and measurements

This retrospective, cross-sectional, single-center study took place over a six-month period from January to July 2016. The charts of patients who had been discharged or died during this study period of interest were reviewed for HIV status. In order to find relevant cases, physicians and research assistants involved in the study examined the daily discharge and death folders on each internal medicine ward in order to capture all eligible patient records.

For patients found to be HIV-positive, researchers completed a standardized data collection form from the patient chart. The form included demographic information, such as age, sex, and date of admission; presenting symptoms and physical exam findings; information about HIV diagnosis, including known or new diagnosis, HAART history, and HIV clinical staging; laboratory data, when available; and final diagnoses and outcomes (death or discharge). Only prerecorded data available in the patient charts were used for data collection, and no patients or physicians were interviewed for data collection.

### Definition of HIV positive status and HAART experience

Patients were considered eligible for review if they were diagnosed with serologically-proven HIV upon current admission to the hospital or if a known prior diagnosis had been documented in the patient chart. A diagnosis was documented as “new” if the patient was diagnosed on current admission to KATH, while a diagnosis was deemed “known” if the patient had ever been previously diagnosed with HIV, even if that diagnosis occurred at a referral hospital just before arriving at KATH. Patients were considered to have HAART experience if the patient’s chart documented that the patient had ever previously been started on HAART or was currently receiving HAART therapy.

### Diagnostic methods

Pulmonary tuberculosis was diagnosed when the patient exhibited (1) consistent clinical findings, (2) suggestive chest x-ray, and (3) placement on standard TB treatment. Patients who presented with focal or generalized neurological symptoms suggestive of an intracranial space-occupying lesion unable to obtain head CT were classified as “undifferentiated intracranial space occupying legion (ICSOL)” with a wide differential diagnosis including stroke, toxoplasmosis, brain abscess, tuberculoma or intracranial malignancy. Patients able to undergo head CT were categorized as CT-suggested toxoplasmosis when radiographic evidence revealed multiple, ring-enhancing lesions. Anemia, thrombocytopenia, and pancytopenia were confirmed via laboratory results in the patient chart. A diagnosis of cryptococcal disease was based on a record of serum or CSF cryptococcal antigen result or Indian ink stain result. Other, less common diagnoses listed in patient charts utilized a variety of clinical and laboratory findings to diagnose the patient.

### Statistical analysis

All patient records used for the study were assigned a random alpha-numeric identifier in order to protect patient confidentiality. Patient records were entered into a FileMaker 12 Pro (FileMaker, Inc., Santa Clara, California) database. Subsequently, data were extracted from the database and analyzed using SAS version 9.4 (Cary, NC).

Summary frequencies and proportions were used to describe the sample for all nominal characteristics including patients’ sex, transfer from another hospital to KATH, symptoms, and comorbidities. Medians with interquartile range were used to describe all other demographics including patients’ age, months since HIV diagnosis, and laboratory values. Univariable Cox proportional hazards models were used to estimate the risk of mortality following date of admission as a function of patients’ comorbidities and demographic characteristics. In these models, elapsed time was measured in days from date of admission to date of death (if deceased) and living patients were censored on their discharge date. The proportional hazard assumption for each predictor was assessed graphically using Martingale residuals as described by Lin, Wei, and Ying [23].

A multivariable Cox proportional hazards model was used to estimate the adjusted risk of mortality as a function of HAART while controlling for patients’ age, sex, presence of neurological symptoms, clinical stage, ICSOL diagnosis, and pneumonia. These covariates were selected because of their importance on univariable analysis and improvement in multivariable model fit statistics, including Akaike information criterion (AIC).

### Ethics clearance

Ethics approval was granted by the Kwame Nkrumah University of Science and Technology and the Komfo Anokye Teaching Hospital ethics review committee (CHRPE/347/15). Consent from individual patients was waived, as this project involved only a retrospective review of patient charts without any acquisition of identifying patient information.

## Results

During the six-month study period, 250 HIV-positive patients were admitted to the KATH internal medicine service and 245 of these individuals had valid survival time recorded. Of these patients, 145/245 (59.2%) were female. Median age of patients included in the study was 42 years old (IQR 35–51). Mortality rate during admission was 35.5% (87 patients). One hundred and forty five (59.4%) of the patients admitted had a known history of HIV documented in their patient chart, while the remaining patients were found to have a new diagnosis of HIV during their admission (Table 1).

**Table 1:** Demographic characteristics and presenting complaints of patients presenting to KATH from January–July 2016

	Valid N	Total
Female Sex	245	145 (59.2%)
*Mdn* Age (IQR)	244	42.0 (35.0–50.5)
*Mdn* Months since HIV diagnosis (IQR)	205	0.82 (0.23–12.29)
Referred to KATH	189	92 (48.7%)
Constitutional Symptoms	240	65 (27.1%)
Neurological Symptoms	239	102 (42.7%)
Pulmonary Symptoms	240	81 (33.8%)
Cardiac symptoms	240	22 (9.2%)
Gastrointestinal Symptoms	241	58 (24.1%)
Other admission reason	243	15 (6.2%)
Known History of HIV	244	145 (59.4%)
HAART	237	99 (41.8%)
Clinical Stage	244	
Stage 0		1 (0.4%)
Stage 1		20 (8.2%)
Stage 2		3 (1.2%)
Stage 3		112 (45.9%)
Stage 4		108 (44.3%)
*Mdn* Hemoglobin (IQR)	230	8.65 (6.90–10.90)
*Mdn* Platelet Count (IQR)	224	269.5 (149.0–377.0)

Unless otherwise noted, valid counts are provided with column proportions. *Mdn* Median, *IQR* Interquartile range. *HAART* Highly active antiretroviral therapy, *HIV* Human immunodeficiency virus, *KATH* Komfo-Anokye Teaching Hospital

There was a broad spectrum of patient complaints at the time of admission. Charts were examined for chief presenting complaint and categorized according to the main body system involved in the complaint (Table 1). Pulmonary and neurologic symptoms predominated among presenting patient complaints, with 33.8% and 42.7% of patients experiencing these complaints respectively. A variety of diagnoses were discovered within this patient population (Table 2).

**Table 2:** Final diagnoses of HIV-positive patients admitted to KATH

	Valid N	Discharged (*N* = 158)	Deceased (*N* = 87)	Total (*N* = 245)
ICSOL	245	28 (17.7%)	26 (29.9%)	54 (22.0%)
Pulmonary Tuberculosis	245	52 (32.9%)	47 (54.0%)	99 (40.4%)
HIV encephalopathy	244	4 (2.5%)	6 (6.9%)	10 (4.1%)
Bacterial Pneumonia	245	20 (12.7%)	21 (24.1%)	41 (16.7%)
Gastroenteritis	245	17 (10.8%)	8 (9.2%)	25 (10.2%)
Candidiasis	245	33 (20.9%)	15 (17.2%)	48 (19.6%)
Pleural Effusion	245	8 (5.1%)	6 (6.9%)	14 (5.7%)
Hypertension	245	20 (12.7%)	5 (5.7%)	25 (10.2%)
Anemia	245	59 (37.3%)	42 (48.3%)	101 (41.2%)
HIV Associated Nephropathy	245	5 (3.2%)	4 (4.6%)	9 (3.7%)
Hepatitis B	239	7 (4.4%)	6 (6.9%)	13 (5.3%)
Unspecified Diarrhea	245	14 (8.9%)	5 (5.7%)	19 (7.8%)

Valid counts are provided with column proportions. *ICSOL* Suspected intracranial space occupying lesion, *HIV* Human immunodeficiency virus, Only diagnoses occurring with *N* ≥ 10 patients are included in the Table. “Discharged” and “deceased” columns are listed with column proportions

As expected, pulmonary tuberculosis predominated among diagnostic findings, with 40.4% of admitted patients diagnosed with the condition during the course of their stay. Based on neurological findings on exam, 54 (22.0%) of patients were suspected to have an intracranial space occupying lesion (ICSOL). However, only 19 (35.2%) of these patients received a head CT scan. Fourteen of the 19 patients (73.7%) able to undergo head CT scan revealed radiographic evidence of toxoplasmosis (multiple, ring-enhancing lesions). Causes of mortality were varied. Of the 87 patients who died while admitted, 54.0% had pulmonary tuberculosis, while 29.9% had a clinical or radiological diagnosis of ICSOL. Another 29.1% were diagnosed with bacterial pneumonia. Other diagnoses are listed in Table 2.

In this sample of data, there was no meaningful difference in time to death between those receiving and not receiving HAART (*p =* 0.89) (see Table 3).

**Table 3:** Univariable analysis of risk of mortality as a function of patient comorbidities and characteristics

	Valid N	Hazard Ratio	95% Confidence Interval		*p*
			Lower	Upper	
Age (per 5 year increase)	244	1.105	0.999	1.222	.052
Male vs Female	245	1.475	0.963	2.258	.07
Referred to KATH: Yes vs No	189	0.828	0.514	1.333	.44
Reason for Admission (Yes vs No)					
Constitutional symptoms	240	0.832	0.512	1.353	.46
Neurological symptoms	239	2.113	1.362	3.279	.001
Pulmonary symptoms	240	0.892	0.566	1.407	.62
Cardiac symptoms	240	0.871	0.433	1.753	.70
Gastrointestinal symptoms	241	0.538	0.301	0.962	.04
Other reason for admission	243	1.576	0.783	3.171	.20
Months since HIV diagnosis (per 1 month increase)	205	1.004	0.998	1.009	.18
New vs Known HIV diagnosis	244	0.866	0.554	1.353	.53
HAART: Yes vs No	237	0.969	0.625	1.502	.89
Prophylaxis Medication: Yes vs No	235	1.146	0.675	1.945	.61
HgB (per 1 unit increase)	230	0.996	0.964	1.029	.81
PLT (per 5 unit increase)	224	1.003	0.996	1.010	.43
Febrile Exam: Yes vs No	232	1.342	0.845	2.132	.21
Clinical Stage (vs Stage 0–2)	244				.12
Stage 3		2.937	0.707	12.191	.14
Stage 4		3.794	0.918	15.680	.07
Final Diagnoses (Yes vs No)					
ICSOL	245	1.724	1.084	2.742	.02
ICSOL with CT	56	0.402	0.162	1.000	.0501
CT-suggestive Toxoplasmosis	245	0.553	0.174	1.756	.32
Pulmonary TB	245	1.041	0.670	1.619	.86
Encephalopathy	244	2.133	0.927	4.909	.07
Pneumonia	245	1.787	1.089	2.931	.02
Gastroenteritis	245	1.217	0.586	2.527	.60
Candidiasis	245	0.939	0.536	1.643	.82
Pleural Effusion	245	0.735	0.317	1.701	.47
HTN	245	0.610	0.246	1.511	.29
Anemia	245	1.139	0.744	1.743	.55
HIV-Associated nephropathy	245	1.331	0.487	3.640	.58
Hepatitis B	245	1.175	0.511	2.702	.70
Diarrhea	245	1.001	0.404	2.478	.99

On univariable analysis, neurological symptoms were associated with an increased risk of mortality. Compared to patients without neurological symptoms, those presenting with neurological symptoms as their reason for admission were 2.11 (95% CI: 1.36–3.28) times more likely to die (*p* = .001). Conversely, patients with gastrointestinal symptoms were less likely to die (*HR* = 0.54, 95% CI: 0.30–0.96), though it is important to note that the upper end of the confidence interval is approaching a null conclusion (*p* = .04). Regarding final diagnosis, patients with an ICSOL diagnosis were 1.72 (95% CI: 1.08–2.74) times more likely to die when compared to patients without an ICSOL diagnosis (*p* = .02), and patients with a diagnosis of pneumonia were 1.79 (95% CI: 1.09–2.93) times more likely to die when compared to patients without a pneumonia diagnosis (*p* = .02). After controlling for patients’ use of HAART as well as their age, sex, clinical stage, ICSOL diagnosis, and pneumonia, those presenting with neurological symptoms remained at high risk for mortality (*HR* = 2.14, 95% CI: 1.27–3.61; *p* = .004) (Table 4).

**Table 4:** Risk of mortality as a function of multivariable patient comorbidities and characteristics

	Adjusted Hazard Ratio	95% Confidence Interval		*p*
		Lower	Upper	
HAART (Yes vs No)	0.997	0.639	1.555	.99
Age (per 5 year increase)	1.050	0.939	1.173	.39
Male vs Female	1.394	0.890	2.185	.15
Neurological symptoms (Yes vs No)	2.138	1.267	3.608	.004
Clinical Stage (vs Stage 0–2)				.27
Stage 3	3.261	0.777	13.686	.11
Stage 4	2.895	0.671	12.489	.15
ICSOL (Yes vs No)	0.966	0.503	1.854	.92
Pneumonia (Yes vs No)	1.435	0.817	2.519	.21

Number of events = 81. Overall model significance *p* = .03

## Discussion

The data collected from this study can aid in the development of strategies to reduce the morbidity and mortality of individuals with HIV infection in Ghana. It has identified the need for additional case finding and screening for HIV diagnosis. This study revealed a large number of newly diagnosed cases of HIV, with 40% receiving a new diagnosis during their current admission to the hospital. While some of these patients may have received a previous diagnosis that they chose not to disclose to the physician, there are likely a high number of new cases among patients that had not been recently screened. Thus, there is a need for increased HIV screening at the local level to enroll patients in treatment in order to achieve 90–90-90 targets [19] in the upcoming years.

The results of this study suggest that tuberculosis represents a major comorbidity (40.4%) amongst HIV-positive patients in this region of Ghana. This high prevalence of tuberculosis is similar to another study examining causes of morbidity at other sites in West Africa [4]. Additionally, neurological complaints commonly occurred in this patient population, with 22.0% of presenting patients thought to have an intracranial space occupying lesion on exam. Among these patients, less than half received CT imaging to confirm diagnosis representing a significant diagnostic barrier. This could contribute to the statistically significant association of neurological symptoms with mortality in the region. The possible reasons for not receiving CT imaging are multifactorial and would include high cost to the patient, lack of health insurance coverage for CT imaging, patient refusal, unavailability of CT imaging machines at the facility and mortality prior to receiving imaging during the inpatient hospital stay. Regional differences in causative organisms for meningitis were also highlighted, with five patients presenting with CSF findings consistent with tuberculosis meningitis (data not shown), while no patients had a positive test result recorded on serum or CSF for *Cryptococc*al infection. This finding exhibited the geographic variability of cryptococcal disease, which is common in eastern Africa [10] but does not appear to be prevalent in Ghana [13].

The results of this study suggest that resource allocation for HIV treatment in this region may be most useful in primary detection of HIV in order to prevent late patient presentation in stage 3 or 4 disease. Tuberculosis diagnosis and treatment must continue to be emphasized, as nearly half of patients diagnosed with pulmonary tuberculosis (47.5%) did not survive until discharge, while over half (54.0%) of all inpatient deaths were related to a tuberculosis diagnosis. Lastly, more resources must be allocated to diagnostic imaging for neurological complaints in HIV patients, as CT scanning is an essential component of diagnosis to differentiate symptoms concerning for intracranial lesions.

There are several potential weaknesses of the study. First, as data collection involved paper chart data retrieval, there are potential missed cases, as charts may have been removed from the ward before data collection was complete. Secondly, misdiagnosis is a possibility, as not all patients were able to undergo necessary investigations to confirm diagnoses, instead undergoing presumptive treatment. This also argues for resource funding from HIV aid groups for necessary diagnostic testing to guide treatment. Patients tested for HIV were screened using antibody testing, meaning some positive patients tested may have been missed during the window period. Lastly, there may have been patients admitted who did not undergo HIV testing or did not disclose their HIV-positive status who were nevertheless HIV-positive.

An additional benefit of this study involves the development of a low-cost, easily manageable database of de-identified patient information that could be used as a model for data collection in other resource-limited hospitals. This model could be expanded to collect data regarding any number of patient populations, not only the HIV-positive population. This study can serve as a foundation for further research to investigate the causes of the observed clinical presentations in this cohort of hospitalized patients—such as the role of patients’ level of access to HIV diagnostic testing and HAART and the prevalence of treatment failure, default, and non-adherence. Ultimately, such research will improve the lives of individuals in Ghana and continue to curtail the HIV epidemic.

## Conclusions

In this population of HIV-positive patients presenting for care at a tertiary care center in Ghana, mortality rate was 35.5%, while 40.6% of patients received a new diagnosis of HIV on admission. Pulmonary tuberculosis was a major comorbidity, while neurological symptomology was a predictor of mortality. These findings argue for greater primary case detection, as well as allocation of resources for imaging and treatment of neurological conditions in HIV-positive patients.

## Abbreviations

HAART: Highly Active Antiretroviral Therapy
HIV: Human Immunodeficiency Virus
ICSOL: Intracranial Space Occupying Lesion
KATH: Komfo Anokye Teaching Hospital
UNAIDS: Joint United Nations Programme on HIV/AIDS
WHO: World Health Organization
